# Timing and intensity of physical activity and late sleeping habits among children in Japan

**DOI:** 10.3389/fped.2022.915758

**Published:** 2022-09-13

**Authors:** Yusuke Matsuyama, Aya Isumi, Satomi Doi, Ai Shibata, Kaori Ishii, Koichiro Oka, Takeo Fujiwara

**Affiliations:** ^1^Department of Global Health Promotion, Tokyo Medical and Dental University, Tokyo, Japan; ^2^Japan Society for the Promotion of Science, Tokyo, Japan; ^3^Faculty of Health and Sport Sciences, University of Tsukuba, Tsukuba, Japan; ^4^Faculty of Sport Sciences, Waseda University, Tokorozawa, Japan

**Keywords:** physical activity, sleep, child, school, accelerometer

## Abstract

**Background:**

Little is known about what timing and intensity of physical activity (PA) are beneficial to preventing children’s late sleeping habits. We investigated the association between timing and intensity of PA and late sleeping habits among Japanese children.

**Methods:**

The amount of PA on a weekday (light (>1.5 to <3.0 metabolic equivalents [METs]); moderate (3.0 to <6.0 METs); and vigorous (6.0 to <20.0 METs) was measured for the whole day, before school, during school, and after school, using accelerometers for population-based fourth-grade elementary school and eighth graders for 7 consecutive days between September and December 2018. Late sleeping habit (going to bed after 10 p.m. for fourth graders and after 11 p.m. for eighth graders) was assessed by questionnaires. The data of 229 fourth graders and 182 eighth graders were analyzed with Poisson regression adjusted for grade, gender, household income, body mass index (BMI), belonging to a sports club, wake-up time on weekdays, and PAs with different intensities.

**Results:**

Total PA was not associated with late sleeping habits. Light-intensity PA before school was protectively associated with late sleeping habits (prevalence ratio [PR]: 0.82, 95% confidence interval [CI]: 0.68, 0.99) while PA at school or after school was not.

**Conclusion:**

Light-intensity PA before school may enhance the earlier bedtime of children.

## Introduction

Sleep plays an essential role in children’s health and wellbeing (1). For example, child adiposity is more prevalent in those with short sleep duration (2). Sleep with poor quality or short duration increases daytime sleepiness and can reduce memory and school performance of children (3). Further, short sleep duration is associated with the psychosocial wellbeing of children, such as emotional regulation (4). Thus, the sleep habits of children could be an important target of public health intervention to improve child health and wellbeing.

The association between physical activity (PA) and the sleep habits of children has been reported, but the results are inconsistent. A study of children aged 3–7 years old in New Zealand reported that children who went to bed later did more sedentary and light-intensity PA but not more moderate-to vigorous-intensity PA than those with earlier bedtimes (5). Another study in Sweden reported that moderate-to vigorous-intensity PA during the day promoted sleep efficiency at night but not total sleeping hours among children aged 6–10 years old (6). In contrast, Pesonen et al. reported an association between PA during the day and decreased sleep duration and increased sleep fragmentation at night (7). Although these studies employed accelerometers to objectively measure the PAs of children, they did not consider the time of the day, or timing when children engaged in PAs, which might have accounted for the inconsistent results. To the best of our knowledge, no studies have investigated the association between timing and intensity of PAs and late sleeping habit among children.

In Japan, there are no national guidelines regarding the sleep of children, neither of appropriate duration nor timing on activity for better sleep. The average sleep hours of children aged 10–14 years old are reportedly 8 h (8), which is shorter than the National Sleep Foundation’s recommendation of 9–11 h for school children (9). Japanese children spent about 4–7 h or more at school on weekdays. Thus, if PA is related to late sleeping habits, the school could be an effective intervention field to enhance the PA of children. In Japan, it is common for some children to go to school earlier so that they can play on the school field before class and participate in PAs at school sports clubs after school hours. Thus, it is worthwhile to investigate the timing and intensity of PA during school, which may prevent late sleeping habits.

This study aimed to investigate the association between the timing of PA, measured with different intensities, before, during, and after school on weekdays, and the sleep habits of children in Japan.

## Materials and methods

### Study participants

**FIGURE 1:**
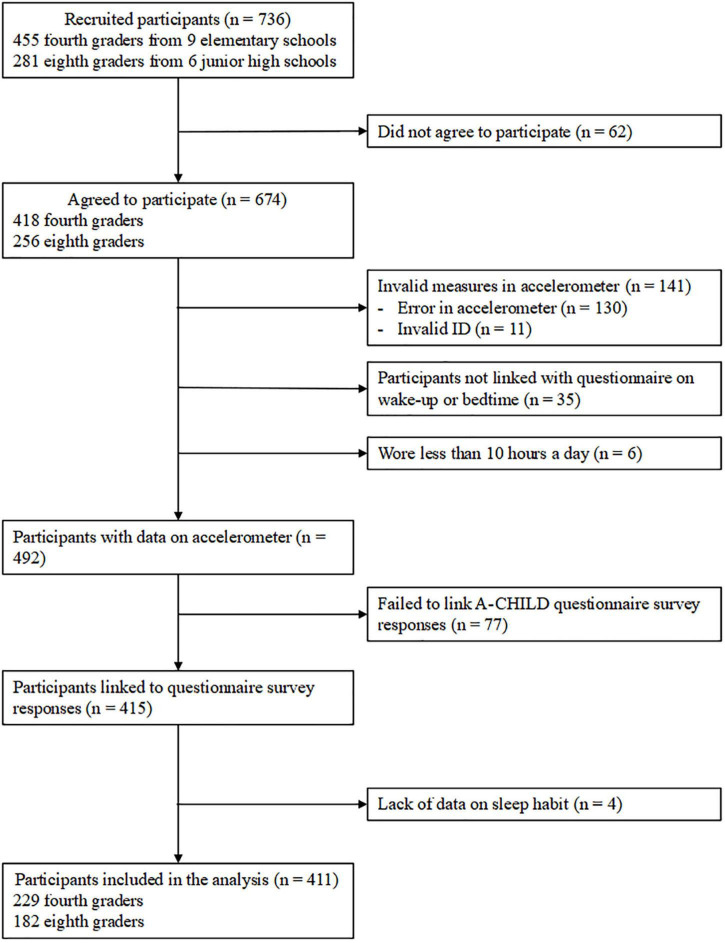
Flow chart of study participants.

This study is a part of the Adachi Child Health Impact of Living Difficulty (A-CHILD) study, a cohort study to investigate the association between socioeconomic factors, such as poverty, lifestyle, and health of school children, in Adachi City, Tokyo, Japan (10). In the present study, data on PA were measured with a tri-axial accelerometer, the Active Style Pro HJA-750C [Omron Healthcare, Kyoto, Japan (11)], which is a new version of the HJA-350IT, a frequently used research-grade tri-axial accelerometers (12). The data were linked to the questionnaire survey conducted in 2018. As shown in Figure 1, 455 fourth graders (i.e., 9–10 years old) in nine public elementary schools and 281 eighth graders (i.e., 13–14 years old) in six public junior high schools are asked to wear an accelerometer on their waist for 7 consecutive days between September and December 2018. These schools were selected by the local city government with consideration for representativeness in terms of social and geographical environments. The children were also asked *via* the questionnaire about their waking time and bedtime during the 7 days. Of the 736 children, 674 children (418 fourth graders and 256 eighth graders) agreed to participate (participation rate: 91.6%). After excluding children with invalid data on accelerometers, such as running out of accelerometer battery, errors when extracting data from the device to the database, and mismatched identification numbers, a valid data set of 492 children was available. As a considerable number of children were excluded due to the unexpected errors when measuring their PA, we did not exclude children by the number of days that they wore an accelerometer. Therefore, the data of all children who wore an accelerometer for 10 h or more during at least a weekday were used. We further excluded children who did not agree to link to the questionnaire survey and those with a lack of information on sleep habits. Finally, 411 children (229 fourth graders and 182 eighth graders) were included in the analysis. The present study was approved by the Ethics Committee at Tokyo Medical and Dental University (M2016-284-02).

### Demographic characteristics

Information on the child’s gender, household income (<3.0, 3.0–5.9, 6.0–9.9, ≥1.0 million JPY, and do not know/do not want to answer; 1 USD = 110 JPY in 2018) was assessed *via* a questionnaire for caregivers. Children were asked whether they belong to any sports clubs (yes/no) and their wake-up time on weekdays (before or after 7 am). Furthermore, following the WHO Child Grows Standards (13), age- and sex-specific body mass index (BMI) z-scores were derived from children’s height and weight measured in school health checkups. The variable on BMI z-scores was categorized to three groups (<−1 SD, −1 SD < +1 SD, and ≥ +1 SD). These variables were used as covariates in the analysis.

### Physical activity on weekdays

Physical activity on weekdays was assessed with accelerometers (HJA-750C), which were distributed *via* the schools. Participants were asked to wear it on their waist for 7 consecutive days between September and December 2018. Epoch length was set at 10 s to account for the young children’s instantaneous activity (14). School starting and ending times of each day were obtained from the schools, and total PA on a weekday that falls within the following timeframes was calculated: (1) after getting up but before starting class (hereafter, before school), (2) during school time, and (3) after school and before bedtime (hereafter, after school). Sedentary behavior (0.9–1.5 metabolic equivalents, METs), light (>1.5 to <3.0 METs), moderate (3.0 to <6.0 METs), and vigorous (6.0 to <20.0 METs) intensities of PA were measured in each timeframe. The conversion equations for children were applied to consider the potential overestimation of MET values among children (15). Consecutive zero counts for 60 min were considered non-wear time, and data were excluded if the total wearing time for the day was less than 10 h (16).

### Late sleeping habit

Habitual bedtime during weekdays was assessed with a questionnaire in the A-CHILD study conducted in October 2018. Children indicated the time they go to bed on weekdays by selecting from the following options: “before 8 p.m.,” “8–9 p.m.,” “9–10 p.m.,” “10–11 p.m.,” “11 p.m.–0 a.m.,” and “0 a.m. or after.” After considering the distribution of the answers, fourth graders with bedtime at 10 p.m. or later and eighth graders with bedtime at 11 p.m. or later were considered to have late sleeping habits.

### Statistical analysis

The association between the average duration of PA per day with different intensities (i.e., sedentary, light, moderate, and vigorous) for each timeframe (before school, during school, and after school) and the late sleeping habits were investigated by Poisson regression analysis adjusted for grade, gender, household income, BMI z-score, belonging to sports clubs, and wake-up time on weekdays. Furthermore, to consider potential confounding among PAs with different intensities, light, moderate, and vigorous PAs at the same time of the day were mutually adjusted. Sedentary behavior was not included in model 2, as we had focused on PAs that could be enhanced by potential interventions at school. PA variables were standardized within the grade.

## Results

Table 1 shows the demographic characteristics of the participants and the amount of PA with different intensities in each timeframe. Of the 411 children, 187 (45.5%) children were boys; 31.9 and 34.1% of the households had 3.0–5.9 and 6.0–9.9 million JPY; 65% were within ± 1 SD of WHO standard BMI z-score; 70.6% belonged to sports club; and 55.7% woke up before 7 a.m. on weekdays.

**TABLE 1:** Demographic characteristics of the study participants (*N* = 411).

		Grade	
	Total	Grade 4	Grade 8
	*N* = 411	*N* = 229 (55.7%)	*N* = 182 (44.3%)
	
	N (%)	N (%)	N (%)
Sex			
Boys	187 (45.5%)	106 (46.3%)	81 (44.5%)
Girls	224 (54.5%)	123 (53.7%)	101 (55.5%)
**Household income (million JPY)**			
<3.0	34 (8.3%)	17 (7.4%)	17 (9.3%)
3.0–5.9	131 (31.9%)	72 (31.4%)	59 (32.4%)
6.0–9.9	140 (34.1%)	81 (35.4%)	59 (32.4%)
≥1.0	56 (13.6%)	33 (14.4%)	23 (12.6%)
Do not know/do not want to answer	50 (12.2%)	26 (11.4%)	24 (13.2%)
**WHO standard BMI z-score**			
< -1SD	80 (19.5%)	35 (15.3%)	45 (24.7%)
≥ -1SD, <1SD	267 (65.0%)	144 (62.9%)	123 (67.6%)
≥ 1SD	59 (14.4%)	49 (21.4%)	10 (5.5%)
Missing	5 (1.2%)	1 (0.4%)	4 (2.2%)
**Belonging to sports club**			
Yes	290 (70.6%)	163 (71.2%)	127 (69.8%)
No	120 (29.2%)	65 (28.4%)	55 (30.2%)
Missing	1 (0.2%)	1 (0.4%)	0 (0.0%)
**Waking-up time on weekdays**			
Before 7 a.m.	229 (55.7%)	130 (56.8%)	99 (54.4%)
7 a.m. or after	182 (44.3%)	99 (43.2%)	83 (45.6%)

SD, standard deviation; BMI, body mass index.

**FIGURE 2:**
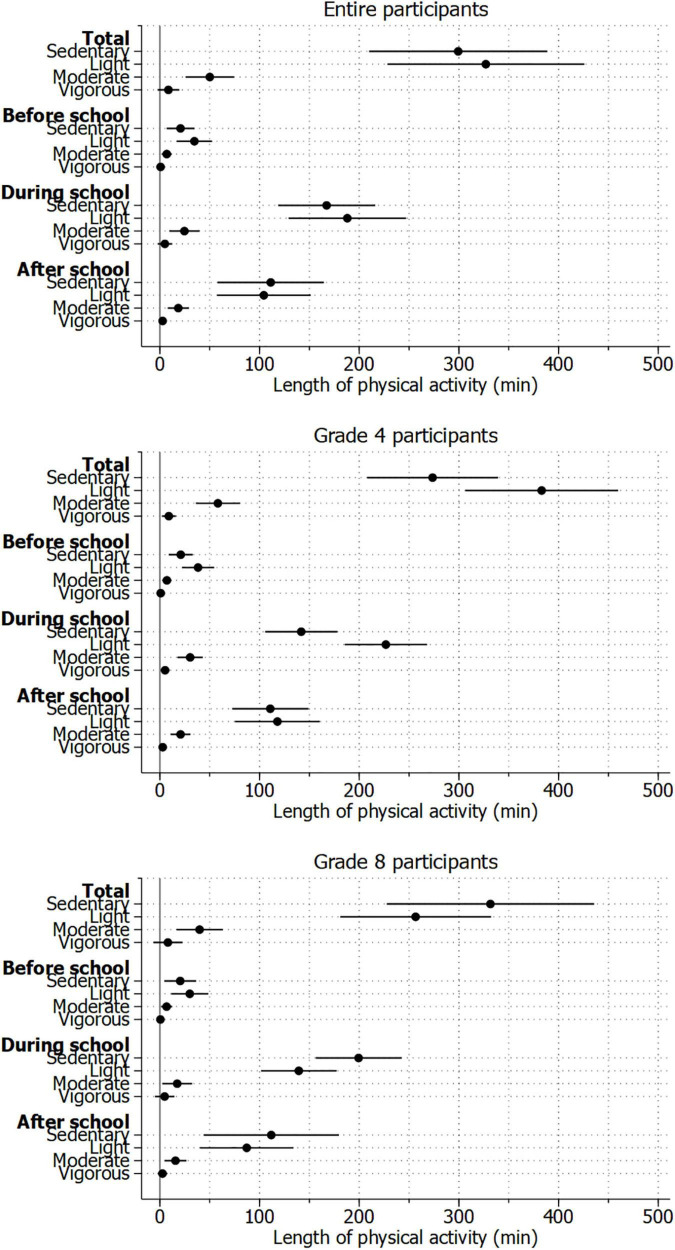
Length of physical activity in a week among the study participants.

Figure 2 shows the duration of PA of children on a weekday. The average sedentary time, light, moderate, and vigorous PAs were 299.3, 327.0, 50.1, and 8.6 min per day, respectively. The duration of PA was the longest during school time, followed by after school and before school. The fourth graders were more physically active than the eighth graders. The average sedentary time, light, moderate, and vigorous intensities of PA for fourth graders were 273.6, 383.0, 58.2, and 9.0 min per day, respectively; that for eighth graders were 331.6, 256.6, 39.9, and 8.1 min per day, respectively.

Table 2 shows the average PA by grade and sleeping habit. Of the 229 fourth graders, 93 (40.6%) had late sleeping habits, while 117 (64.3%) of the 182 eighth graders had one too. Children without a late sleeping habit showed longer sedentary time, light, and moderate intensities of PA before school. Vigorous-intensity PA before school and other intensities during or after school were not significantly different from having late sleeping habits.

**TABLE 2:** Average physical activity by grade and sleeping habit.

	Grade 4			Grade 8		
	Going to bed before 10 p.m.	Going to bed after 10 p.m.		Going to bed before 11 p.m.	Going to bed after 11 p.m.	
	*N* = 136 (59.4%)	*N* = 93 (40.6%)		*N* = 65 (35.7%)	*N* = 117 (64.3%)	
		
	Mean (SD)	Mean (SD)	*P*-value	Mean (SD)	Mean (SD)	*P*-value
**PA total (min)**						
Sedentary	272.9 (62.6)	274.7 (70.1)	0.840	333.8 (111.7)	330.4 (99.9)	0.830
Light	386.8 (73.0)	377.5 (81.8)	0.370	264.2 (78.2)	252.3 (74.1)	0.310
Moderate	58.6 (21.9)	57.6 (22.5)	0.730	43.2 (23.7)	38.1 (22.8)	0.150
Vigorous	9.2 (7.3)	8.7 (6.5)	0.540	8.9 (14.1)	7.6 (14.7)	0.570
**PA before school (min)**						
Sedentary	23.7 (12.3)	17.1 (10.6)	< 0.001	26.7 (19.4)	17.0 (12.5)	< 0.001
Light	42.1 (15.4)	32.9 (15.6)	< 0.001	35.7 (22.4)	26.8 (15.2)	0.002
Moderate	7.6 (4.4)	6.3 (5.1)	0.045	7.9 (5.7)	6.2 (5.4)	0.041
Vigorous	1.0 (1.6)	0.7 (0.9)	0.092	0.6 (1.0)	0.6 (1.6)	0.930
**PA during school (min)**						
Sedentary	140.4 (35.6)	144.0 (37.1)	0.460	197.2 (42.3)	200.6 (43.7)	0.600
Light	225.4 (40.9)	228.7 (41.9)	0.560	139.6 (38.9)	139.3 (37.4)	0.960
Moderate	30.3 (13.2)	30.5 (12.0)	0.900	18.7 (17.3)	16.7 (13.5)	0.400
Vigorous	5.5 (4.8)	4.9 (4.1)	0.360	5.7 (10.7)	4.3 (9.2)	0.370
**PA after school (min)**						
Sedentary	108.8 (35.4)	113.6 (42.0)	0.350	110.0 (70.6)	112.8 (66.5)	0.790
Light	119.3 (39.5)	116.0 (46.7)	0.560	88.9 (44.6)	86.2 (48.2)	0.710
Moderate	20.8 (8.8)	20.8 (11.3)	0.980	16.6 (10.5)	15.2 (11.2)	0.390
Vigorous	2.8 (2.3)	3.0 (2.8)	0.390	2.6 (4.0)	2.7 (5.4)	0.960

SD, standard deviation; PA, physical activity.

Sedentary: 0.9–1.4 metabolic equivalents, METs; light: 1.5–2.9 METs; moderate: 3.0–5.9 METs; vigorous: 6.0–19.9 METs.

**FIGURE 3:**
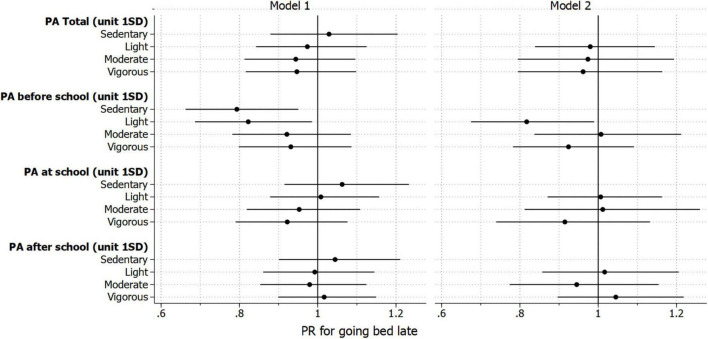
Association between physical activity (PA) and late sleeping habit; late sleeping habit was defined as 10 p.m. or later for fourth graders and 11 p.m. or later for eighth graders; sedentary behavior: 0.9–1.5 METs; light: >1.5 to <3.0 METs; moderate: 3.0 to <6.0 METs; vigorous: 6.0 to <20.0 METs; model 1 was adjusted for grade, gender, household income, body mass index, belonging to a sports club, and wake-up time; each physical activity variable was separately included; model 2 was further mutually adjusting for light, moderate, and vigorous intensities of physical activity in the same timing of the day.

Figure 3 shows the association of PA by intensity and timeframe with late sleeping habits. Total PA per day was not associated with the late sleeping habits of children. After adjusting for grade, gender, household income, BMI z-score, belonging to sports clubs, and wake-up time on weekdays, sedentary behavior before school showed a significant inverse association with late sleeping habits [prevalence ratio (PR): 0.79, 95% confidence interval (CI): 0.66, 0.95]. Light-intensity PA before school showed a significant inverse association with late sleeping habits (PR: 0.82, 95% CI: 0.69, 0.99). In the model that mutually adjusted for light, moderate, and vigorous PAs in the same timeframe of the day, light-intensity PA showed a significant inverse association with late sleeping habits (PR: 0.82, 95% CI: 0.68, 0.99). On the contrary, vigorous-intensity PA before school was not significantly associated with the late sleeping habits of children. Furthermore, PA of any intensity during or after school was not significantly associated with the late sleeping habits of children. The detailed numbers are shown in Supplementary Table 1.

## Discussion

This study is the first to report the association between PA according to intensity and timing of the day and late sleeping habits among children. The analysis found that light-intensity PA before school was protectively associated with late sleeping habits, which is not true for moderate or vigorous intensity of PA before school or PA of any intensity during or after school.

It is worth comparing the amount of PA between the participants of the present study and those of another similar study. A study reported that sedentary behavior, light, moderate, and vigorous intensities of PA of children in Okayama City, Japan were 420, 318, 51, and 29 min, respectively, for higher grades of elementary school, and 520, 252, 27, and 20 min, respectively, for junior high school students (17). The participants of the present study had a similar amount of light- and moderate-intensity PA but less sedentary behavior and vigorous-intensity PA. This difference could be attributed to the differences in the study field, that is, geographical location (Adachi City is located in urban Tokyo), age of children, and the make of the accelerometer (one by Lifecorder Suzuken Co., was used in the study).

Some studies have investigated the association between PAs and the sleep habits of children. Williams et al. reported that children aged 3–7 years old with more sedentary behavior and light-intensity PA had later sleeping time (5). Similarly, a multinational study of 12 countries found that children aged 9–11 years with more sedentary behavior had later bedtime, while those with the more moderate-to-vigorous intensity of PA had earlier sleeping time (18). In contrast, Ekstedt et al. reported that elementary school children with more moderate-to-vigorous intensity PA had later bedtime, while no significant association was found with total sleep time (6). These inconsistent results suggest a complex nature of the relationship between PA and the bedtime of children. Importantly, none of these studies have considered the time of the day when the children participated in PA.

The present study found no association between total PA per day with any intensity of PA and late sleeping habits. However, participating in light-intensity PA before school was associated with having earlier sleep habits. These findings, in turn, suggest a potential program for children to have better sleep. According to the present study’s estimate, the prevalence of children with late sleeping habits was decreased by about 18 percentage points per 1 SD (i.e., 10–20 min) increment in light-intensity PA before school. Engaging in light-intensity PA, such as walking on the school field or stretching in the classroom before class, may improve the child’s sleep habits. As for the inverse association between sedentary behavior before school and having late sleeping habits, it might suggest that those who wake up early but do not participate in any PA tend to go to bed early. However, it would be difficult for schools to implement a program to enhance sedentary behavior, which has been shown to be associated with poor health outcomes in children (19). Further study is needed to consider the more detailed information on children’s behavior before going to school.

This study has limitations. First, the sleep habit of children was self-reported. Sleep outcomes could be detected from accelerometer measurements; however, the algorithm is still under development (20). In addition, the definition of late bedtime was not predefined but is based on the distribution of the responses. Second, a considerable number of children was excluded due to invalid data on accelerometers; however, most of the excluded cases were due to empty batteries or errors in extracting data from the device to the database, which are unlikely to be affected by the children’s PA, and thus this exclusion would not lead to severe bias. Third, this is a cross-sectional study, and the causality between PA and sleep habits is unknown, i.e., children with late sleeping habits might be less likely to participate in light-intensity PA. Fourth, all the study participants were from the same city in Tokyo. Their PA patterns might differ from children in other areas in Japan, especially those in rural areas because the environment of the neighborhood and school have an influence on a child’s PA (21).

## Conclusion

In conclusion, the present study found an inverse association between light-intensity PA before school and late sleeping habits among school children in Tokyo, Japan. Encouraging PA before school might improve children’s sleep. A future longitudinal study is required to confirm the finding.

## Data availability statement

The datasets presented in this article are not readily available due to patients data privacy. Requests to access the datasets should be directed to the corresponding author.

## Ethics statement

The studies involving human participants were reviewed and approved by the Ethics Committee at Tokyo Medical and Dental University. Written informed consent to participate in this study was provided by the participants’ legal guardian/next of kin.

## Author contributions

YM contributed to the analysis and interpretation and drafted manuscript. AI and SD contributed to the data acquisition, project administration, interpretation, and critically revised manuscript. AS, KI, and KO contributed to the conception and design, interpretation, and critically revised manuscript. TF contributed to the conception and design, data acquisition, funding acquisition, project administration, interpretation, and critically revised manuscript. All authors gave final approval and agreed to be accountable for all aspects of the work.

## References

[1] Matricciani L Paquet C Galland B Short M Olds T. Children’s sleep and health: a meta-review. *Sleep Med Rev.* (2019) 46:136–50. 31121414 10.1016/j.smrv.2019.04.011

[2] FelsÖ R Lohner S Hollódy K Erhardt É Molnár D. Relationship between sleep duration and childhood obesity: systematic review including the potential underlying mechanisms. *Nutr Metab Cardiovasc Dis.* (2017) 27:751–61. 28818457 10.1016/j.numecd.2017.07.008

[3] Dewald JF Meijer AM Oort FJ Kerkhof GA Bögels SM. The influence of sleep quality, sleep duration and sleepiness on school performance in children and adolescents: a meta-analytic review. *Sleep Med Rev.* (2010) 14:179–89. 20093054 10.1016/j.smrv.2009.10.004

[4] Chaput J-P Gray CE Poitras VJ Carson V Gruber R Olds T Systematic review of the relationships between sleep duration and health indicators in school-aged children and youth. *Appl Physiol Nutr Metab.* (2016) 41:S266–82. 27306433 10.1139/apnm-2015-0627

[5] Williams SM Farmer VL Taylor BJ Taylor RW. Do more active children sleep more? A repeated cross-sectional analysis using accelerometry. *PLoS One.* (2014) 9:e93117. 10.1371/journal.pone.0093117 24695112 PMC3973701

[6] Ekstedt M Nyberg G Ingre M Ekblom Ö Marcus C. Sleep, physical activity and BMI in six to ten-year-old children measured by accelerometry: a cross-sectional study. *Int J Behav Nutr Phys Act.* (2013) 10:82. 10.1186/1479-5868-10-82 PMC3691618 23800204

[7] Pesonen A-K Sjöstén NM Matthews KA Heinonen K Martikainen S Kajantie E Temporal associations between daytime physical activity and sleep in children. *PLoS One.* (2011) 6:e22958. 10.1371/journal.pone.0022958 21886770 PMC3160292

[8] Cabinet Office *Kodomo-Wakamono Hakusho [in Japanese]* Tokyo: Cabinet Office (2015).

[9] Hirshkowitz M Whiton K Albert SM Alessi C Bruni O DonCarlos L National Sleep Foundation’s sleep time duration recommendations: methodology and results summary. *Sleep Health.* (2015) 1:40–3. 29073412 10.1016/j.sleh.2014.12.010

[10] Ochi M Isumi A Kato T Doi S Fujiwara T. Adachi Child Health Impact of Living Difficulty (A-CHILD) study: research protocol and profiles of participants. *J Epidemiol.* (2021) 31:77–89. 32201401 10.2188/jea.JE20190177 PMC7738641

[11] Yano S Koohsari MJ Shibata A Ishii K Frehlich L McCormack GR Comparison of older and newer generation active style pro accelerometers in physical activity and sedentary behavior surveillance under a free-living environment. *Int J Environ Res Public Health.* (2019) 16:1597. 10.3390/ijerph16091597 PMC6539210 31067688

[12] Ohkawara K Oshima Y Hikihara Y Ishikawa-Takata K Tabata I Tanaka S. Real-time estimation of daily physical activity intensity by a triaxial accelerometer and a gravity-removal classification algorithm. *Br J Nutr.* (2011) 105:1681–91. 21262061 10.1017/S0007114510005441

[13] de Onis M Onyango AW Borghi E Siyam A Nishida C Siekmann J. Development of a WHO growth reference for school-aged children and adolescents. *Bull World Health Organ.* (2007) 85:660–7. 18026621 10.2471/BLT.07.043497 PMC2636412

[14] Aadland E Andersen LB Anderssen SA Resaland GK Kvalheim OM. Accelerometer epoch setting is decisive for associations between physical activity and metabolic health in children. *J Sports Sci.* (2020) 38:256–63. 31735120 10.1080/02640414.2019.1693320

[15] Tanaka C Reilly JJ Tanaka M Tanaka S. Seasonal changes in objectively measured sedentary behavior and physical activity in Japanese primary school children. *BMC Public Health.* (2016) 16:969. 10.1186/s12889-016-3633-5 27618879 PMC5020446

[16] Aadland E Andersen LB Anderssen SA Resaland GK. A comparison of 10 accelerometer non-wear time criteria and logbooks in children. *BMC Public Health.* (2018) 18:323. 10.1186/s12889-018-5212-4 29510709 PMC5840816

[17] Ishii K Shibata A Adachi M Nonoue K Oka K. Gender and grade differences in objectively measured physical activity and sedentary behavior patterns among Japanese children and adolescents: a cross-sectional study. *BMC Public Health.* (2015) 15:1254. 10.1186/s12889-015-2607-3 26679503 PMC4683705

[18] Chaput J-P Katzmarzyk PT LeBlanc AG Tremblay MS Barreira TV Broyles ST Associations between sleep patterns and lifestyle behaviors in children: an international comparison. *Int J Obes Suppl.* (2015) 5:S59–65. 27152187 10.1038/ijosup.2015.21 PMC4850622

[19] WHO. *WHO Guidelines on Physical Activity and Sedentary Behaviour.* (2020). Available online at: https://www.who.int/publications/i/item/9789240015128 (accessed August 4, 2022). 33369898

[20] van Kooten JAMC Jacobse STW Heymans MW de Vries R Kaspers GJL van Litsenburg RRL A meta-analysis of accelerometer sleep outcomes in healthy children based on the Sadeh algorithm: the influence of child and device characteristics. *Sleep.* (2021) 44:zsaa231. 10.1093/sleep/zsaa231 33161428

[21] Mayne SL Mitchell JA Virudachalam S Fiks AG Williamson AA. Neighborhood environments and sleep among children and adolescents: a systematic review. *Sleep Med Rev.* (2021) 57:101465. 10.1016/j.smrv.2021.101465 PMC8164975 33827031

