# Supplementary material for: Timing and intensity of physical activity and late sleeping habits among children in Japan
Source: Front Pediatr. 2022 Sep 13;10:915758. doi: 10.3389/fped.2022.915758 (PMC9513050; doi:10.3389/fped.2022.915758)
Supplement: Supplementary file 1 [file Table_1.docx]

Supplementary Table S1. Estimates for the association between physical exercise before, at, after school, and late sleeping habit

|  | Crude model | | Grade and gender adjusted | | Model 1 | | Model 2 | |
| --- | --- | --- | --- | --- | --- | --- | --- | --- |
|  | PR (95% CI) | P-value | PR (95% CI) | P-value | PR (95% CI) | P-value | PR (95% CI) | P-value |
| PA total (unit 1 SD) |  |  |  |  |  |  |  |  |
| Sedentary | 1.00 (0.87,1.15) | 0.990 | 0.99 (0.85,1.16) | 0.946 | 1.03 (0.88,1.20) | 0.722 |  |  |
| Light | 0.94 (0.82,1.07) | 0.359 | 0.93 (0.81,1.07) | 0.333 | 0.97 (0.84,1.12) | 0.716 | 0.98 (0.84,1.14) | 0.796 |
| Moderate | 0.94 (0.82,1.08) | 0.412 | 0.94 (0.81,1.09) | 0.410 | 0.94 (0.81,1.10) | 0.449 | 0.97 (0.79,1.19) | 0.798 |
| Vigorous | 0.96 (0.83,1.11) | 0.567 | 0.96 (0.82,1.12) | 0.581 | 0.95 (0.82,1.10) | 0.470 | 0.96 (0.79,1.16) | 0.686 |
| PA before school (unit 1 SD) |  |  |  |  |  |  |  |  |
| Sedentary | 0.73 (0.63,0.86) | <0.001 | 0.71 (0.60,0.84) | <0.001 | 0.79 (0.66,0.95) | 0.012 |  |  |
| Light | 0.76 (0.66,0.88) | <0.001 | 0.74 (0.63,0.87) | <0.001 | 0.82 (0.69,0.99) | 0.034 | 0.82 (0.68,0.99) | 0.039 |
| Moderate | 0.85 (0.73,1.00) | 0.051 | 0.85 (0.72,1.00) | 0.047 | 0.92 (0.78,1.08) | 0.325 | 1.01 (0.84,1.21) | 0.939 |
| Vigorous | 0.94 (0.80,1.09) | 0.409 | 0.93 (0.80,1.10) | 0.416 | 0.93 (0.80,1.09) | 0.365 | 0.92 (0.78,1.09) | 0.353 |
| PA during school (unit 1 SD) |  |  |  |  |  |  |  |  |
| Sedentary | 1.04 (0.91,1.20) | 0.537 | 1.05 (0.90,1.21) | 0.544 | 1.06 (0.92,1.23) | 0.426 |  |  |
| Light | 1.02 (0.89,1.17) | 0.782 | 1.02 (0.89,1.17) | 0.774 | 1.01 (0.88,1.16) | 0.907 | 1.01 (0.87,1.16) | 0.931 |
| Moderate | 0.98 (0.85,1.12) | 0.756 | 0.98 (0.84,1.14) | 0.786 | 0.95 (0.82,1.11) | 0.531 | 1.01 (0.81,1.26) | 0.918 |
| Vigorous | 0.94 (0.81,1.09) | 0.385 | 0.93 (0.80,1.09) | 0.390 | 0.92 (0.79,1.08) | 0.304 | 0.91 (0.74,1.13) | 0.414 |
| PA after school (unit 1 SD) |  |  |  |  |  |  |  |  |
| Sedentary | 1.04 (0.91,1.19) | 0.548 | 1.04 (0.90,1.21) | 0.558 | 1.04 (0.90,1.21) | 0.564 |  |  |
| Light | 0.97 (0.85,1.11) | 0.641 | 0.96 (0.83,1.11) | 0.593 | 0.99 (0.86,1.14) | 0.918 | 1.02 (0.86,1.21) | 0.851 |
| Moderate | 0.97 (0.85,1.12) | 0.708 | 0.98 (0.85,1.12) | 0.723 | 0.98 (0.85,1.12) | 0.767 | 0.95 (0.77,1.15) | 0.580 |
| Vigorous | 1.03 (0.91,1.17) | 0.643 | 1.03 (0.91,1.17) | 0.623 | 1.02 (0.90,1.15) | 0.794 | 1.04 (0.90,1.22) | 0.575 |

Abbreviation: standard deviation, SD; physical activity, PA

Sedentary behavior: 0.9–1.5 METs; light: >1.5 to <3.0 METs; moderate: 3.0 to <6.0 METs; vigorous: 6.0 to <20.0 METs

PA variables were standardized within the grade

Model 1: adjusted for grade, gender, household income, body-mass index, belonging to sports club, and wake-up time; each physical activity variable was separately included

Model 2: Model 1 + mutually adjusting for light, moderate, and vigorous intensity of physical activity in the same timing of the day
